# Overexpression of an Acidic Endo-β-1,3-1,4-glucanase in Transgenic Maize Seed for Direct Utilization in Animal Feed

**DOI:** 10.1371/journal.pone.0081993

**Published:** 2013-12-31

**Authors:** Yuhong Zhang, Xiaolu Xu, Xiaojin Zhou, Rumei Chen, Peilong Yang, Qingchang Meng, Kun Meng, Huiying Luo, Jianhua Yuan, Bin Yao, Wei Zhang

**Affiliations:** 1 Biotechnology Research Institute, Chinese Academy of Agricultural Sciences, Beijing, P. R. China; 2 Key Laboratory for Feed Biotechnology of the Ministry of Agriculture, Feed Research Institute, Chinese Academy of Agricultural Sciences, Beijing, P. R. China; 3 Institute of Food Crops, Jiangsu Academy of Agricultural Sciences, Nanjing, P. R. China; TGen, United States of America

## Abstract

**Background:**

Incorporation of exogenous glucanase into animal feed is common practice to remove glucan, one of the anti-nutritional factors, for efficient nutrition absorption. The acidic endo-β-1,3-1,4-glucanase (Bgl7A) from *Bispora* sp. MEY-1 has excellent properties and represents a potential enzyme supplement to animal feed.

**Methodology/Principal Findings:**

Here we successfully developed a transgenic maize producing a high level of Bgl7AM (codon modified Bgl7A) by constructing a recombinant vector driven by the embryo-specific promoter ZM-leg1A. Southern and Western blot analysis indicated the stable integration and specific expression of the transgene in maize seeds over four generations. The β-glucanase activity of the transgenic maize seeds reached up to 779,800 U/kg, about 236-fold higher than that of non-transgenic maize. The β-glucanase derived from the transgenic maize seeds had an optimal pH of 4.0 and was stable at pH 1.0–8.0, which is in agreement with the normal environment of digestive tract.

**Conclusion/Significance:**

Our study offers a transgenic maize line that could be directly used in animal feed without any glucanase production, purification and supplementation, consequently simplifying the feed enzyme processing procedure.

## Introduction

β-1,3-1,4-d-Glucans (β-glucans) are the main component of cereal cell walls, particularly in the endosperm cell walls of barley and other grains [1]. It is composed of β-d-glycosyl residues linked through irregular β-1,3 and/or β-1,4 glycosidic bonds. Ruminants can utilize β-glucans through enzyme digestion of rumen microbes. However, monogastric animals such as pig, poultry, and fish do not have such enzymes to decompose the β-glucans. By combining with water, β-glucans increase the viscosity of chyme, block the intestinal surface partially, and prevent the mixing of intestinal endogenous digestive juice with the chyme [2]. Thus β-glucan represents one of the intense anti-nutritional factors in wheat- and barley-based diets [3].

To overcome these problems, the most common and effective practice is to add exogenous endoglucanases into animal feed [3]. Majority of endoglucanases are grouped into glycoside hydrolase (GH) families 3, 5, 7, 12 and 16, based on the amino acid sequence and catalytic domain structures (http://www.cazy.org/). According to the degradation mode against glycosidic linkage, endoglucanases have been grouped into four main categories: β-1,3-glucanase (laminarinase, EC 3.2.1.39), β-1,4-glucanases (cellulase, EC 3.2.1.4), β-1,3-1,4-glucanases (lichenase, EC 3.2.1.73), and β-1,3(4)-glucanase (EC 3.2.1.6) [4]. Among them, β-1,3-1,4-glucanase has received significant attention in feed industrial applications because of their hydrolysis ability against grain-based glucan and multiple enzymatic functions. β-1,3-1,4-Glucanase is able to catalyze the hydrolysis of β-glucan into low molecular weight glucose polymers, thus reducing the hydrophilicity and viscosity of chyme and eliminating the anti-nutritional negative effect. Moreover, addition of β-1,3-1,4-glucanase can improve feed intake, enhance animal production, regulate cecal microbiota and increase feed conversion ratio [5]–[8]. Besides, the hydrolysis products from glucans—glucooligosaccharides may serve as fermentable dietary fiber-like substrates and positively affect gastrointestinal tract health [9].

To date, commercial feed additive β-1,3-1,4-glucanases are generally from microbial expression systems, commonly *Aspergillus japonicus* [10], *Pichia pastoris* [11] and *Clostridium thermocellum* [8]. This process is flexible and convenient, but has disadvantages like high energy consumption, high equipment cost and serious environmental pollution. Moreover, enzyme addition is a complex process involving enzyme isolation, purification and supplementation, which requires more energy and resources. Thus it's a good way to produce feed enzymes (e.g. β-1,3-1,4-glucanase) in transgenic feed grains directly without any industrial processing.

Transgenic plants are being developed for both commercial and environmental values. In 2011, the plantation area of transgenic plants reached about 160 million hectares worldwide and was distributed in 29 countries; transgenic maize accounted for nearly one third of the total genetically modified crops [12]. Maize (*Zea mays* L.) is the main ingredient of animal feed (nearly 50%), and represents an ideal bioreactor of feed enzymes because of its cultivation worldwide. A phytase gene *phyA2* from *Aspergillus niger* has been successfully overexpressed in maize seeds [13].

In this study, we developed a genetically stable maize line that had high β-glucanase activity in the seeds. The endo-β-1,3-1,4-glucanase, Bgl7A, from acidophilic *Bispora* sp. MEY-1 was selected due to its excellent properties as feed additive, such as acidic pH optimum, good thermostability and broad pH stability, highly resistance to proteases, and broad substrate specificity [11]. The gene codon was optimized for better expression in maize.

## Materials and Methods

### Plant materials

Maize Hi-II [14] was used for genetic transformation as host variety. The immature embryos, approximately 1.0–2.0 mm long, were preserved on N61-100-25 medium [14] containing 0.2% (w/v) phytagel (Sigma, St. Louis, MO) for callus induction. The commercial maize inbred-line Zheng58 was used as recurrent parent to produce progenies.

### Codon optimization of the β-1,3-1,4-glucanase gene *bgl7A*

To improve its expression level in transgenic maize, the DNA sequence of native endo-β-1,3-1,4-glucanase gene *bgl7A* from *Bispora* sp. MEY-1 (Genbank accession No. FJ695140) [11] was optimized according to the translationally optimal codon usage of maize [15]. Codon adaptation index (CAI), optimal codon usage, GC content and distribution, effective number of codons (Nc), negative CIS elements, negative repeat elements, and mRNA structure were used to evaluate the gene sequence (https://www.genscript.com/cgi-bin/tools/rare_codon_analysis). Low-usage codons (<15% frequency) were replaced by high-usage ones according to the known codon bias of maize [15]. The modified gene was named *bgl7Am* that encoded the same amino acid sequence as *bgl7A*. The optimized gene was synthesized by Genscript (Nanjing, China) and cloned into pUC57 vector to construct the recombinant plasmid pUC57-*bgl7Am*.

### Plasmid construction

**Figure 1:**
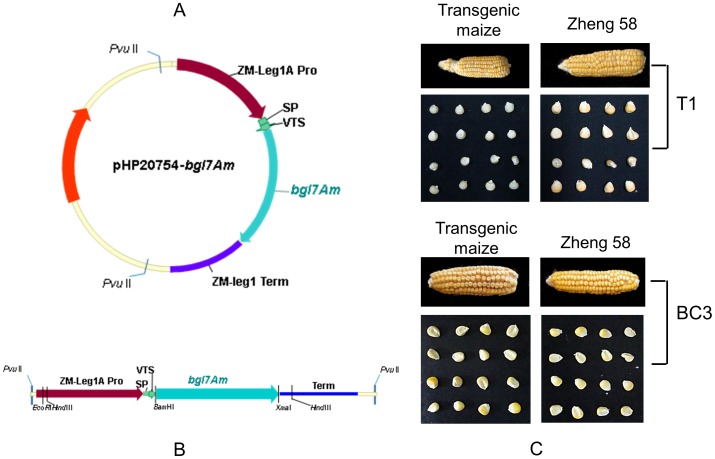
Construction of the recombinant vector and the transgenic maize seed. **A** The recombinant expression vector pHP20754-*bgl7Am*. **B** The chimeric gene cassettes for expression in maize. **C** Ears and seeds of transgenic maize (T1 and BC3) compared with that of wild-type Zheng58.

The transformation vector pHP20754 (Fig. 1a) consists of the corn *legumin1A* (leg1) promoter ZM-leg1A Pro, signal peptide (SP), vacuole targeting sequence (VTS) of corn Proaleurain and the corn leg1 terminator ZM-leg1 Term [16]–[18]. The β-glucanase gene was excised from pUC57-*bgl7Am* with *Bam*HI and *Xma*I and subcloned into pHP20754 to produce the expression construct pHP20754-*bgl7Am*, which was further digested with *Pvu*II to generate the chimeric gene expression cassette (Fig. 1b) for transformation. The plasmid pHP17042BAR carrying the maize histone H2B promoter, the maize Ubiquitin 5′-UTR intron-1, the *bar* gene and the potato protease II (PINII) terminator [13] was used as the selectable marker for screening of positive transgenic plants. The *bar* gene expression cassette was excised from pHP17042BAR by digest with *Hind*III, *Xho*I and *Sac*I.

### Maize transformation, selection and regeneration

The plasmid fragments containing the gene cassette of *bgl7Am* and *bar*, respectively, were mixed at the ratio of 1∶1 and adjusted the concentration to 200 ng/µL. Maize transformation was carried out with high-velocity tungsten microprojectile (Bio-Rad, Hercules, CA) wrapped by the DNAs of *bgl7Am* and *bar* according to the method described before [19]. After recovery, embryonic calli were transferred onto the selective medium supplemented with bialaphos as the selectable marker. The positively transformed calli were cultivated in differentiation medium and rooting medium in succession. Seedlings (T0 plants) were transplanted into greenhouse and pollinated with the inbred-line Zheng58 to produce T1 seeds. Seeds were dried on the plant and harvested 35–45 days after pollination. Zheng58 was used as recurrent parent for backcrossing to produce filial generations (T1, BC1, BC2 and BC3). β-Glucanase activity determination in the kernels and PCR for the *bgl7Am* gene of seedlings were used in combination to screen the transgenic lines.

### PCR detection of exogenous gene integration

The specific primers bgl7am-875F (5′-ACGGCAAGGTCATCCAGAACGCGAAGG-3′) and 20754-398R (5′-TTCCTGGCAAATCACTCGGTGTATC-3′) were used for PCR detection of the positive plants harboring *bgl7Am*. The gene *actin* as control was amplified using primers AC326F (5′-ATGTTTCCTGGGATTGCCGAT-3′) and AC326R (5′-GCATCACAAGCCAGTTTAACC-3′). Genomic DNA of the maize immature leaves was used as PCR templates. The recombinant plasmid pHP20754-*bgl7Am* and the genomic DNA of Zheng58 were used as the positive and negative controls, respectively.

### Southern blot analysis

Five grams of maize leaves of generations T1 to BC3 were ground to powder in liquid nitrogen, and the genomic DNA was extracted with the CTAB method. Genomic DNA of Zheng58 was used as the negative control. About 50 µg of genomic DNA was digested by *Eco*RI and *Hind*III and then separated on a 0.8% (w/v) agarose gel. The agarose gel was transferred onto a hybond-N^+^ nylon membrane (GE Healthcare, Uppsala, Sweden) with a Trans-Blot SD system followed by UV-crosslinking. A digoxin-labeled probe containing a 800-bp fragment of *bgl7Am* was used for southern-blot hybridization. Immunologic process was conducted following the instructions of DIG-high prime DNA labeling and detection starter kit II (Roche, Indianapolis, IN).

### Western blot analysis

Five milligrams of lyophilized purified Bgl7A produced in *Pichia pastoris* GS115 [11] was used for the production of polyclonal antibody in rabbits. Recombinant proteins were extracted from seed meals. Kernels were ground with a high-throughput tissue homogenizer Geno/Grinder 2010 (SEPX CertiPrep, Metuchen, NJ).

To extract protein from seed meals, 30 mg of seed powder were placed into a 1.5-mL tube containing 300 µL extraction buffer (50 mM citric acid-Na_2_HPO_4_, pH 3.5). The tube was agitated on a shaker at room temperature for 1 h. After centrifugation at 5000× *g* for 10 min, the supernatant was incubated with 2-fold volume of pre-cooled acetone for 30 min, followed by centrifugation at 14,000× *g* for 10 min. The protein precipitate was dissolved in 30 µL of deionized water, and the protein sample was divided into two equal parts. One was deglycosylated with endo-β-*N*-acetylglucosaminidase (Endo H) according to the supplier's instructions (New England Biolabs, Ipswich, MA), the other remained intact. Protein extract of purified Bgl7A from *P. pastoris* and Zheng58 were used as the positive and negative controls, respectively. Proteins from the stem, root and leaf of a transgenic plant of generation BC1 were extracted in the same way and used for tissue specificity analysis.

Proteins were separated on SDS–PAGE (12% acrylamide, 0.4% acryl-bisacrylamide). and transferred onto PVDF membrane (Pall, Port Washington, NY). The polyclonal antibody raised in rabbits was added into the membrane confining liquid for prehybridization. The goat anti-rabbit IgG labeled with alkaline phosphatase was used as the secondary antibody. BCIP/NBT kit (Zomanbio, Beijing, China) was used for color development. To identify the proteins, bands were excised from the gel and analyzed using matrix assisted laser desorption/ionization time of flight mass spectrometry (MALDI-TOF-MS) at Tianjin Biochip Corporation (Tianjin, China).

### β-Glucanase activity assay and enzyme characterization

Crude proteins of five randomly selected seeds were extracted with extraction buffer as described above, and the supernatant was subject to β-glucanase activity assay. β-Glucanase activity was determined by measuring the amount of reducing sugar released from lichenan with the method of 3,5-dinitrosalicylic acid (DNS) [11], [20]. One unit of enzyme activity was defined as the amount of enzyme required to release 1 µmol of reducing sugar per minute from 1.0% lichenan in citric acid-Na_2_HPO_4_ (50 mM, pH 3.5) at 60°C for 10 min. β-Glucanase activities of generations T1, BC1, BC2 and BC3 of transgenic maize and Zheng58 were all evaluated. Each reaction and its control were run in triplicate. The enzyme properties of Bgl7AM derived from maize was determined using crude proteins from BC1 seeds as in Luo et al. (2010). The pH optimum of the protein was determined at 60°C and pH 1.0–6.0. The pH stability was determined by measuring the residual activity under standard conditions (pH 5.0, 60°C and 10 min) after pre-incubation at 37°C and pH 1.0–9.0 for 1 h. The optimal temperature was determined at 25–80°C at pH 5.0. Thermal stability of the enzyme was determined by assessing the residual enzyme activity under standard conditions after incubation of the enzyme at 70°C for various durations.

### Evaluation of anti-inactivation stability over feed pelleting process

Feed pelleting was carried out with a twin-screw extruder (DSE-25 Extruder Lab-Station Brabender OHG, Duisburg, Germany). Part of the maize seeds were mixed and extruded at 70°C or 80°C, respectively. β-Glucanase activities and dry matter content (DM) values were determined before and after pelleting. Zheng58 seeds were treated as the non-transgenic control. Stability comparison was conducted with the β-glucanases derived from transgenic maize seeds and *P. pastoris*. Crude Bgl7A derived from *P. pastoris* with equal enzyme activity to transgenic maize was added into Zheng58 seeds, followed by pelleting treatment as described above. And the β-glucanase activity was detected after pelleting. One-way analysis of variance (ANOVA) was performed using the Duncan's multiple-range test to compare treatment means. Significance was defined at P<0.05.

## Results

### Construction and transformation of transgenic vector pHP20754-bgl7Am

The CAI value and GC content of *bgl7A* were 0.715 and 49.6%, respectively. After codon optimization and gene modification, the CAI value and GC content of *bgl7Am* was increased to 0.937 and 67.0%, respectively (Figure S1 and S2). These higher values are better for exogenous gene expression in maize. Furthermore, effective Nc, negative CIS elements, negative repeat elements, and mRNA structure of the target gene were also considered in gene modification (Figure S1, S2 and S3). As a result, native *bgl7A* and synthetic *bgl7Am* shared 82.2% nucleotide sequence identity but encoded identical amino acid sequences.

The 1221-bp *bgl7Am* was inserted into the expression vector pHP20754 between the embryo-specific ZM-leg1A promoter and ZM-leg1 terminator (Fig. 1b), which is a transcriptionally active spacer region that allows highly efficient transgene expression. The positive calli of maize Hi-II were regenerated on bialaphos medium and identified by PCR.

### Plant regeneration and phenotypic evaluation

The regenerated young plants described above showed good growth in the greenhouse. A total of 27 independent transgenic lines were obtained. Based on β-glucanase activities of the seeds, 330 seeds of three independent transgenic events (40, 46 and 51-1) were selected to cultivate in fields and backcross with Zheng58 for progeny production. As shown in Fig. 1c, the ears and seeds of generation T1 showed significant phenotypic difference from Zheng58. This difference was generally subsided in the later generations because of the successive backcrossing with Zheng58. Up to transgenic generation BC3, the traits of transgenic maize were almost the same as that of non-transgenic Zheng58 through visual observation. The result suggests that the inserted exogenous gene has no negative impact on the maize seed.

### Determination of exogenous gene integration

**Figure 2:**
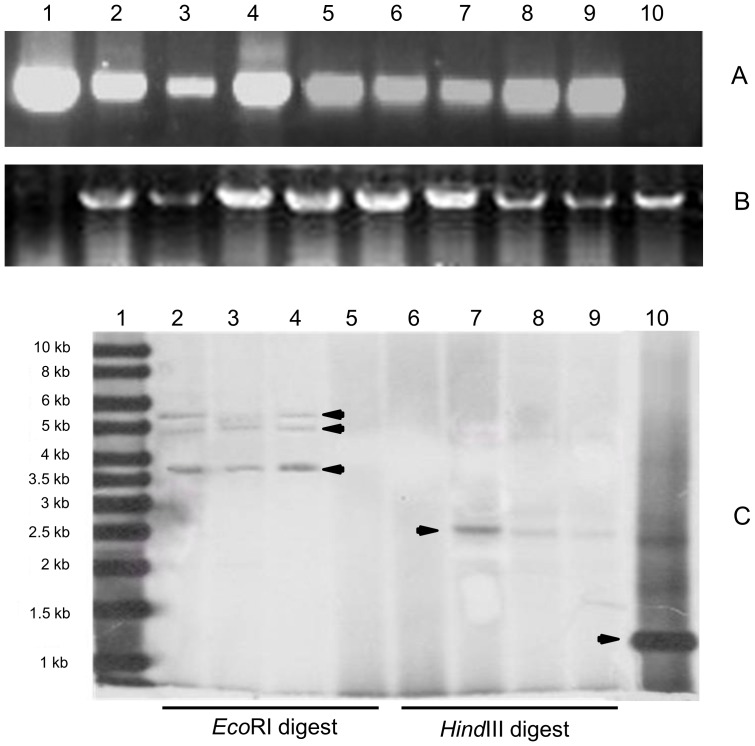
PCR analysis and southern blot analysis of the transgenic maize. **A** PCR detection of the gene *bgl7Am* in the genomic DNA of transgenic plant leaves of generation BC1. Lane 1, the plasmid PHP20754-*bgl7Am* as positive control; lane 2–9, the transgenic plants; lane 10, the non-transgenic Zheng58. **B** PCR detection of the gene *actin*. Lane 1, the plasmid PHP20754-*bgl7Am*; lane 2–9, the transgenic plants; lane 10, the non-transgenic Zheng58. **C** Southern blot analysis of *bgl7Am* in transgenic plants. The *Eco*RI and *Hind*III-digested genomic DNA was hybridized with the *bgl7Am* probe. Lane 1, the DIG-labeled molecular weight markers; lane 2–4, transgenic plants digested by *Eco*RI (arrowhead indicate the positive bands); lane 5, non-transgenic Zheng58 digested by *Eco*RI; lane 6, non-transgenic Zheng58 digested by *Hind*III; lane 7–9, transgenic plants digested by *Hind*III (arrowhead indicate the positive band); lane 10, PCR fragment of the *bgl7Am* as a positive control (arrowhead).

PCR assay with primers specific for *bgl7Am* was used to evaluate the inheritance of transgenic maize. Gene fragments of about 500 bp were detected in the transformation events 40, 46 and 51-1 (Fig. 2a). PCR results of *actin* gene (∼300 bp) indicated the high quality of genomic DNA (Fig. 2b). To confirm the gene integration and the copy number of *bgl7Am* in transgenic plants, the genomic DNAs of three positive transgenic plants of event 40 were analyzed by southern blot after restriction digest with *Eco*RI *and Hind*III. The *bgl7Am* probe was prepared with a 800-bp fragment of the *bgl7Am* gene. There is only one *Eco*RI restriction site located between the promoter and the *bgl7Am* gene in the expression cassette of pHP20754-*bgl7Am*. A total of three bands of ∼3.5, 5.0 and 6.0 kb, respectively, were detected in the positive lane via *Eco*RI digest, but not in non-transgenic Zheng58 (Fig. 2c). There is no *Hind*III site in the *bgl7Am*. While *Hind*III cut the gene expression cassette twice and released an internal fragment of 2.5 kb (Fig. 2c). These results indicate that there are three copies of *bgl7Am* in event 40.

### Evaluation of site-specific expression

**Figure 3:**
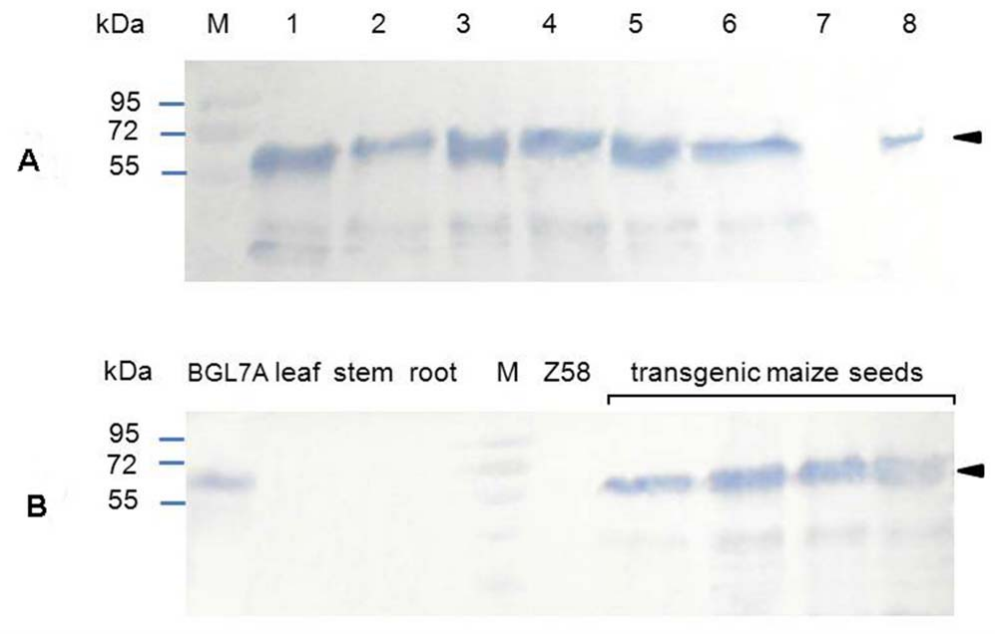
Western blot analysis of recombinant Bgl7AM from the transgenic maize. **A** Western blot analysis of the Bgl7AM from transgenic maize seeds. Lane M, the protein molecular markers; Lane 1, 3, 5, the proteins isolated from transgenic maize seeds; lane 2, 4, 6, the proteins isolated from transgenic maize seeds and treated with Endo H; lane 7, the non-transgenic Zheng58 as a negative control; lane 8, the purified *P. pastoris* Bgl7A as a positive control. **B** Western blot analysis of the Bgl7AM from different tissues of the transgenic maize (leaf, stem, root and seeds). Lane M, the protein molecular markers; purified *P. pastoris* Bgl7A as a positive control; Z58 refers to non-transgenic Zheng58 (negative control).

To determine the expression efficiency of exogenous Bgl7AM, proteins were extracted from two BC1 plants of event 40 that had high β-glucanase activities. In western blot analysis, no band was detected in the negative control of Zheng58 (Fig. 3a). The positive control, Bgl7A expressed by *P. pastoris*, showed a band of about 60 kDa, the same as that reported before [11]. One main band of ∼60 kDa was identified on the PVDF membrane after hybridization with the antibody (Fig. 3a). This molecular weight (60 kDa) was much higher than the predicted molecular weight (45.3 kDa). After Endo H treatment, the band had no significant reduction in molecular weight (Fig. 3a). It suggested that other post-translation modifications rather than *N*-glycosylation, such as *O*-linked glycosylation, phosphorylation, acetylation or methylation, may occur in the transgenic maize. The band was verified to be Bgl7AM through MALDI-TOF-MS analysis (Figure S4). Except for the seeds, proteins extracted from the root, stem and leaf of the positive lines had no objective band (Fig. 3b), indicating the tissue specificity of Bgl7AM. Moreover, Bgl7AM present in seeds are more convenient for storage, transportation and direct utilization.

### Evaluation of seed-derived β-glucanase activity

Positive transgenic plants of transgenic event 40 were selected for β-glucanase activity assay. Approximately 200–400 seeds of each generation were assessed using the DNS method (Table 1). Compared with the non-transgenic Zheng58 that had β-glucanase activity of 3300 U/kg of seeds, T1 seeds (207,800 U/kg) showed approximately 63-fold activities of Zheng58. Both the maximal and average activities of BC1, BC2 and BC3 seeds were increased slightly. The maximal β-glucanase activity of BC3 seeds was up to 779,800 U/kg, which was 236 folds of that of Zheng58. About 47% of the seeds showed over 200,000 U/kg of β-glucanase activity. The result further confirmed that *bgl7Am* is genetically stable over generations in maize.

**Table 1:** β-Glucanase activities of the transgenic maize seeds within four generations.

Generation	Number of seeds with β-glucanase activity (U/kg)*					Maximal activity (U/kg)	Average activity (U/kg)**
	>200,000	100,000–200,000	50,000–100,000	10,000–50,000	<10,000		
T1	2	3	4	90	149	807,800	18,650±1,108^b^
BC1	167	77	11	48	297	734,100	112,700±10,33^c^
BC2	55	14	0	33	317	689,500	167,400±12,54^d^
BC3	103	7	1	11	98	779,800	239,300±8,646^e^
Zheng58	0	0	0	0	13	3,300	780±235^a^

Five kernels from each ear were randomly selected, pooled, and glucanase activity assayed. One unit of enzyme activity was defined as the amount of enzyme required to release 1 µmol of reducing sugar per minute from 1.0% lichenan at 60°C for 10 min. U/kg, glucanase units per kilogram of seed.

The values were means of three replicates±standard deviation.

^a,b,c,d,e^ Means in the same column not sharing a common superscript are significantly different (P<0.05).

### Characterization of maize seed-derived Bgl7AM

**Figure 4:**
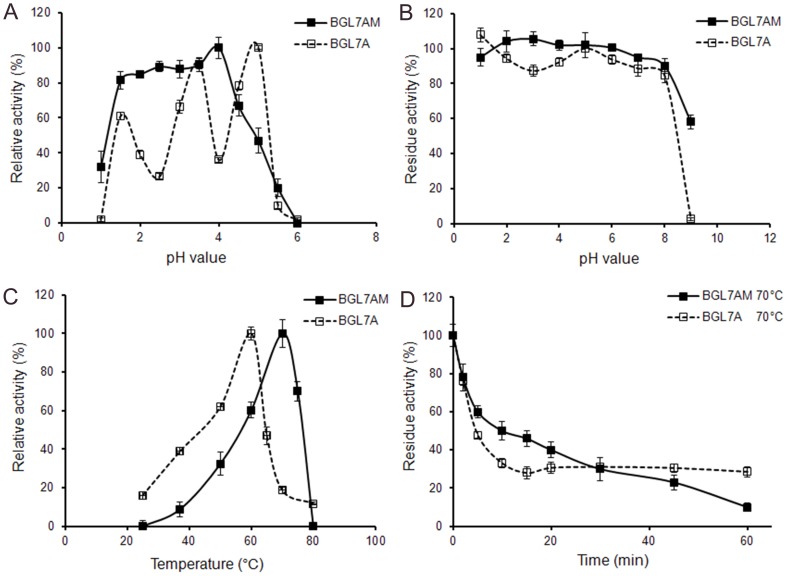
Property comparison of recombinant endo-β-1,3-1,4-glucanase expressed in maize (BGL7AM) and *P. pastoris* (BGL7A). **A** Effect of pH on β-glucanase activity of BGL7AM and BGL7A at 60°C. **B** pH stability of BGL7AM and BGL7A. After incubation at 37°C for 1 h in buffers ranging from pH 1.0 to 9.0, the β-glucanase activity was assayed in 100 mM citric acid-Na_2_HPO_4_ (pH 5.0) at 60°C. **C** Temperature-dependent activity profiles of BGL7AM and BGL7A in 100 mM citric acid-Na_2_HPO_4_ (pH 5.0). **D** Thermostability of BGL7AM and BGL7A pre-incubated at 70°C at pH 5.0. The aliquots were removed at different time points then measure residual β-glucanase activity at 60°C and pH 5.0. Error bars represent the standard deviation of triplicate measurements.

The crude proteins of transgenic BC1 seeds were characterized (Fig. 4), and compared with Bgl7A of *P. pastoris* reported before [11]. Bgl7AM had a pH optimum at 4.0, while Bgl7A exhibited high activity at pH 1.5, 3.5 and 5.0 (maxima). Both enzymes remained active at pH 1.0–8.0. The temperature optimum of Bgl7AM was 70°C, which was 10°C higher than that of Bgl7A. Moreover, thermostability of Bgl7AM was improved. After incubation at 70°C for 15 min, Bgl7AM retained 50% of the initial activity while Bgl7A remained less than 30%.

### Evaluation of anti-inactivation stability in feed pelleting

The β-glucanase activities of Bgl7A (from *P. pastoris*) and Bgl7AM (from transgenic maize seeds) were determined after feed pelleting at 70°C and 80°C, respectively (Table 2). The initial β-glucanase activities in transgenic line or in Zheng 58 by supplementation of Bgl7A were set to 77,860 U/kg and 85,350 U/kg, respectively. After pelleting at each of the tested temperatures, Bgl7A lost more activities than Bgl7AM. In combination with the data of enzyme characterization, Bgl7AM was more excellent than Bgl7A even though they had complete identical amino acid sequences.

**Table 2:** Stability of of Bgl7A and Bgl7AM during feed pelleting*.

	β-Glucanase activity (U/kg seeds)**		Activity loss (%)
	Before pelleting	After pelleting	
70°C			
Bgl7A	85.35±4.43	52.48±3.66	38.51±3.12^b^
Bgl7AM	77.86±6.30	56.82±2.31	27.02±4.83^a^
80°C			
Bgl7A	85.35±3.45	44.21±2.14	48.20±4.31^c^
Bgl7AM	77.86±5.36	49.61±3.22	36.28±3.32^b^

Bgl7A was the recombinant protein expressed in *P. pastoris*; Bgl7AM was the recombinant protein expressed in transgenic maize seeds. The amino acid sequences of Bgl7A and Bgl7AM are totally identical.

The values were means of three replicates±standard deviation.

^a,b,c^ Means in the same column not sharing a common superscript are significantly different (P<0.05).

## Discussion

So far several β-glucanase genes have been introduced into plants for different purposes. Endo-β-1,3-glucanase (laminarinase) can defend plants against fungal pathogens, introduction of its coding genes into crops is a plausible strategy to develop durable resistance against fungal pathogens [21]. Over the last two decades, transgenic plants harboring endo-β-1,4-glucanase (cellulase) genes have taken more attention for conversion of cellulosic biomass into fermentable sugars [22], [23]. Production of recombinant endo-β-1,4-glucanases E1 in transgenic plants have been reported in *Arabidopsis* [24], leaf and root tissues of maize [25], [26] and rice seeds [27]. Endo-β-1,3-1,4-glucanase (lichenase) is an important enzyme additive in monogastric animal feed to decompose β-glucan in cereals [3], [6], [28]. Up to now, β-1,3-1,4-glucanases have been expressed in transgenic barley [29], [30] and potato [31] for feed purpose. However, maize seed has never been used for production of endo-β-1,3-1,4-glucanase. Here we developed a transgenic maize line that overexpressed an endo-β-1,3-1,4-glucanase from *Bispora* sp. MEY-1 in seeds. Compared with enzyme production by microbial fermentation and other transgenic crops, transgenic maize seed has several advantages, such as low cost production, cultivation worldwide and direct utilization in animal feed. On the other hand, the genetic manipulation of maize is more easily. For feed industry's interest, maize seed as the major feed ingredient represents an ideal bioreactor to produce feed enzymes.

About 65% of the maize seed produced in China is used as feed. If maize seeds express sufficient endo-β-1,3-1,4-glucanase, no supplementation of microbial glucanase will be required. To achieve high-level expression of *bgl7Am* in maize seed, several strategies have been utilized in combination, including a synthetic gene with preferred maize codons [15], a strong tissue-specific promoter, and an excellent transformation receptor with high competence and regeneration capacity that improves the transformation efficiency [13], [14]. As a result, the average and maximum glucanase activities in maize seeds without purification and enrichment were up to 239,300 and 779,800 U/kg seeds, respectively (Table 1). Previous feeding trials have shown that the effectiveness of glucanase as a feed additive was maximized at approximately 30,000 units per kg of diets [5]. Typically maize grains constitute 50% of the animal diet, thus the transgenic maize seeds having an average glucanase activity of 239,300 U/kg is high enough to substitute the glucanase supplement. When the transgenic maize line developed in this study is propagated in field, it will enhance the nutritive values of glucan-abundant grains such as wheat and barley. The development of transgenic maize will not only reduce the loss of resources and simplify the production process, but also provide an environmental friendly approach to produce enzymes.

Moreover, Bgl7AM has good thermostability and excellent acidic stability, which are important factors for supplementation to animal feed. Thermostability is a key index of feed enzyme because of the high temperature during feed processing. Since most β-1,3-1,4-glucanases are not stable during coating of feed pellets (70–90°C), selection of a thermostable β-1,3-1,4-glucanase with high activity is of great interest to the animal feedstuff industries [8], [32], [33]. Bgl7AM retains most activities after pelleting at 80°C. This thermostability allows it to survive the heat generated from maize pressing into feed pellets and pasteurization. Similar results that plant-derived enzymes showed better stability have been reported. [34], [35]. This phenomenon might be ascribed to the different folding patterns and disulphide bond formations in microbes and plants [35]. Protection from maize seed starch might be the other cause.

Furthermore, feed enzyme should be stable within the acid environment of monogastric animals' digestive tract, in which the pH value is lower than pH 3.0 in stomach (pH 1.3–3.5 for pigs and pH 2.8–4.8 for chickens) [36]. An acidic-tolerable β-glucanase has been isolated from *Trichoderma koningii* ZJU-T, with optimal activity at pH 2.0 [33]. Molecular modification approaches have been employed to enhance the activity of a β-1,3-1,4-glucanase at acidic pH [37]. Compared with counterparts, Bgl7AM is highly active and stable within pH 1.0–4.0, and retains above 90% of its activity at pH 3.0, the average pH in the animal digestive tract.

This study provides an environment-friendly and low-cost approach to produce transgenic maize with social and ecological significance. It's the first report that produces a biologically active endo-β-1,3-1,4-glucanase in transgenic maize seeds. Approaches to increase the seed glucanase activities are preceding, including selection of more transgenic events and application of stronger promoters. In the future studies, we'll evaluate its direct application effectiveness in animal feed by comparison with traditional feed supplemented with glucanases.

## Supporting Information

Figure S1 **Codon related parameters of wild-type gene** ***bgl7A*** **and optimized** ***bgl7Am*** **.** **A** Codon adaptation index (CAI), negative CIS elements, and negative repeat elements of the *bgl7A* and *bgl7Am*. **B** effective number of codons (Nc) of the *bgl7A* and *bgl7Am.*
(TIF)

Figure S2 **Codon usage and GC content of wild-type gene** ***bgl7A*** **and optimized** ***bgl7Am*** **.** **A** Relative codon frequency of *bgl7A*. **B** Relative codon frequency of *bgl7Am*. **C** GC content and distribution of *bgl7A*. **D** GC content and distribution of *bgl7Am*. **E** Percentage of high frequency used codons of maize in *bgl7A*. **F** Percentage of high frequency used codons of maize in *bgl7Am.*
(TIF)

Figure S3 **mRNA structure prediction of wild-type gene** ***bgl7A*** **and optimized** ***bgl7Am.***
(TIF)

Figure S4 **MALDI-TOF-MS analysis of the BGL7AM from transgenic maize seeds.** **A** Peptide fragments produced by digestion with protease. **B** Analysis of the identified sequence by Mascot.
(TIF)
