# Supplementary figures and images for: Overexpression of an Acidic Endo-β-1,3-1,4-glucanase in Transgenic Maize Seed for Direct Utilization in Animal Feed
Source: PLoS One. 2013 Dec 31;8(12):e81993. doi: 10.1371/journal.pone.0081993 (PMC3876984; doi:10.1371/journal.pone.0081993)

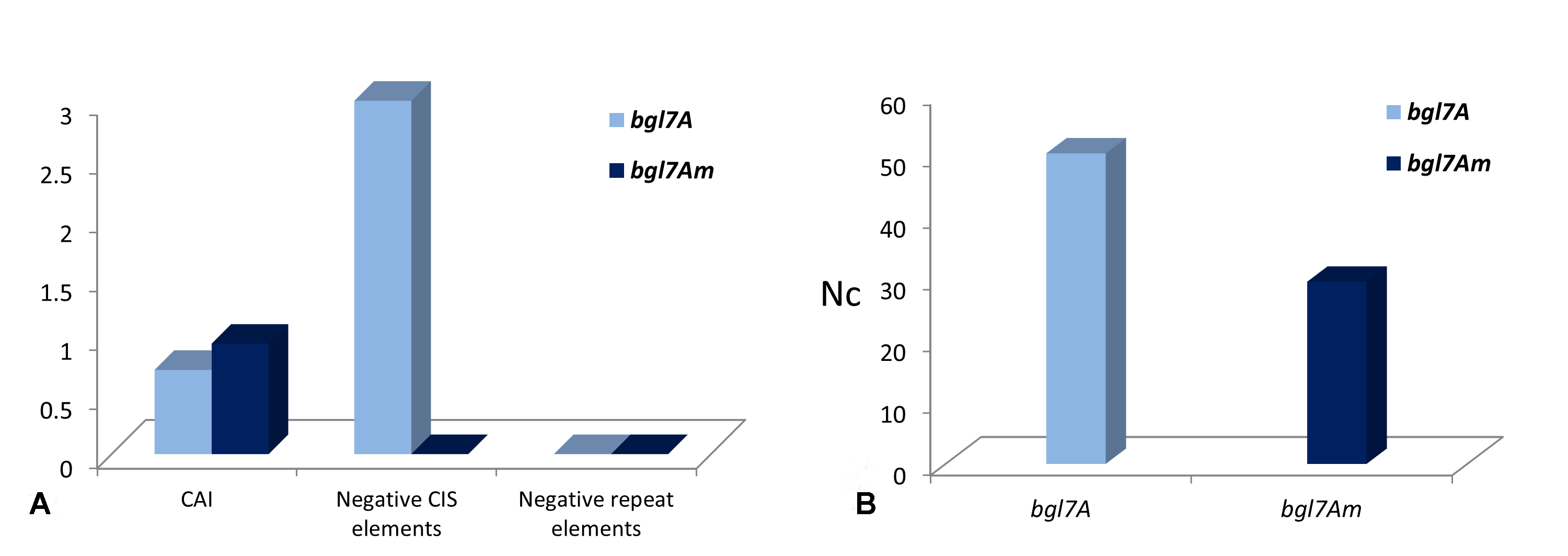

Supplement: Figure S1 — Codon related parameters of wild-type gene bgl7A and optimized bgl7Am . A Codon adaptation index (CAI), negative CIS elements, and negative repeat elements of the bgl7A and bgl7Am. B effective number of codons (Nc) of the bgl7A and bgl7Am. (TIF) [file pone.0081993.s001.tif]

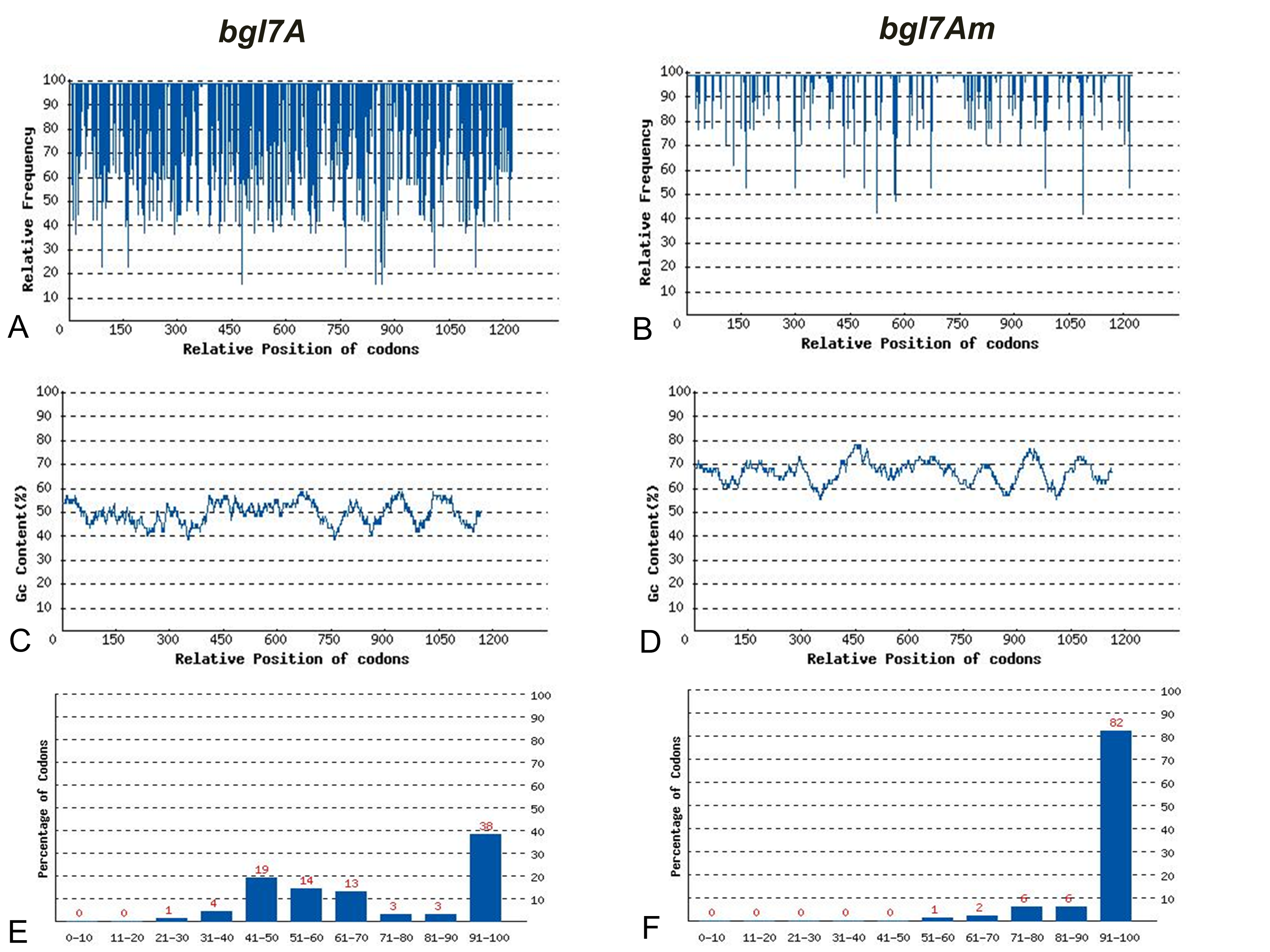

Supplement: Figure S2 — Codon usage and GC content of wild-type gene bgl7A and optimized bgl7Am . A Relative codon frequency of bgl7A. B Relative codon frequency of bgl7Am. C GC content and distribution of bgl7A. D GC content and distribution of bgl7Am. E Percentage of high frequency used codons of maize in bgl7A. F Percentage of high frequency used codons of maize in bgl7Am. (TIF) [file pone.0081993.s002.tif]

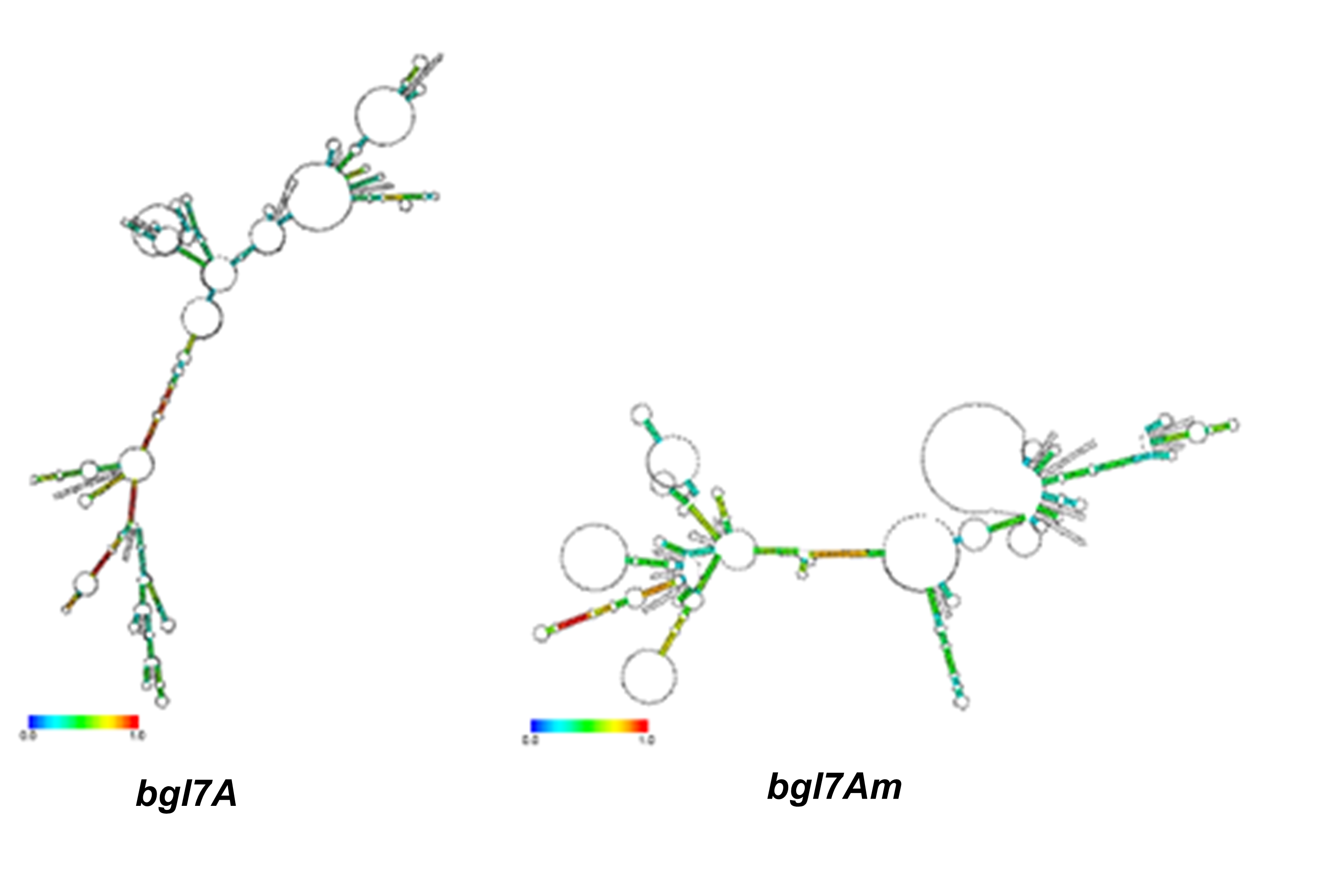

Supplement: Figure S3 — mRNA structure prediction of wild-type gene bgl7A and optimized bgl7Am. (TIF) [file pone.0081993.s003.tif]

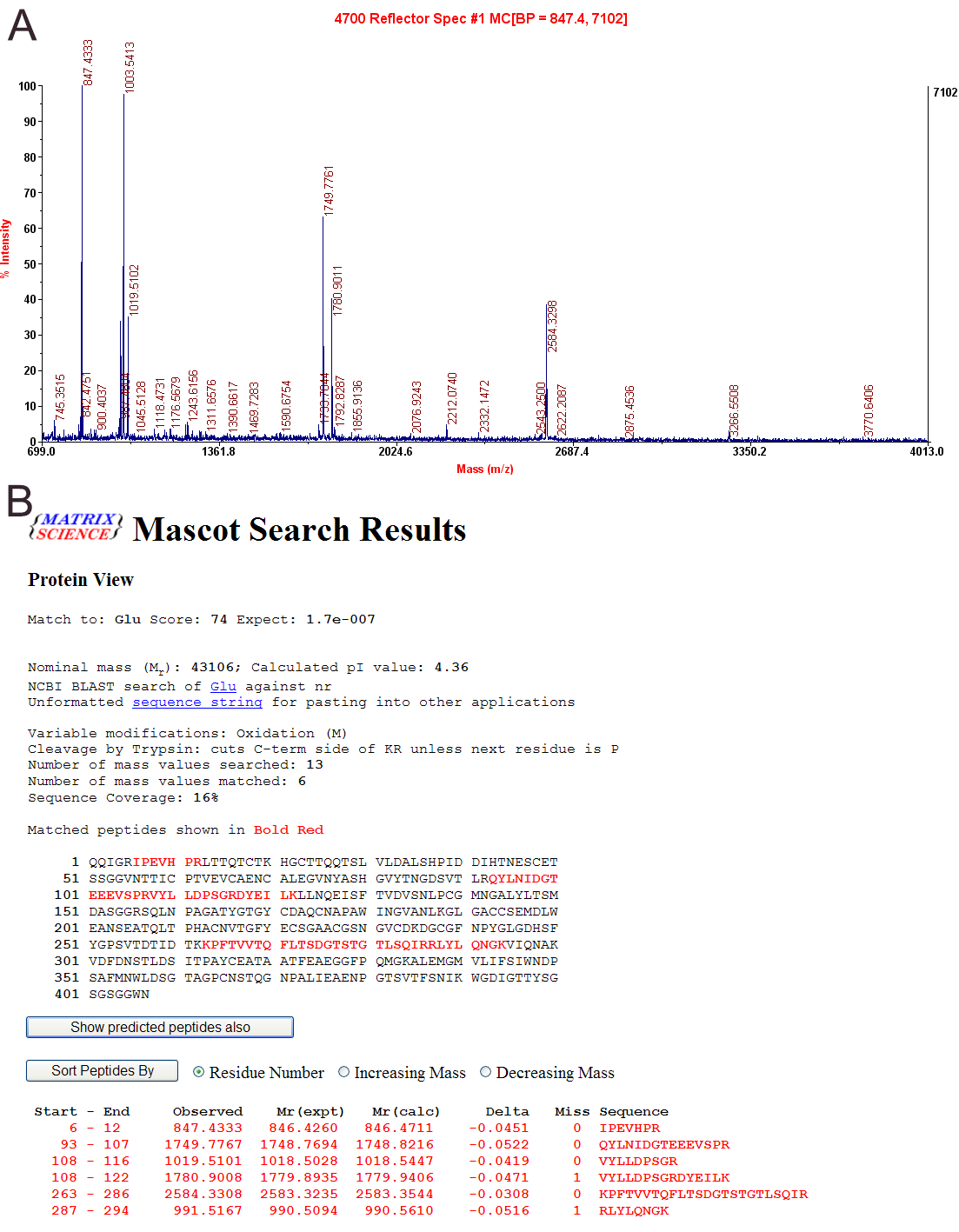

Supplement: Figure S4 — MALDI-TOF-MS analysis of the BGL7AM from transgenic maize seeds. A Peptide fragments produced by digestion with protease. B Analysis of the identified sequence by Mascot. (TIF) [file pone.0081993.s004.tif]
